# Ethyl 4-(5-bromo-2-hy­droxy­phen­yl)-2,7,7-trimethyl-5-oxo-1,4,5,6,7,8-hexa­hydro­quinoline-3-carboxyl­ate

**DOI:** 10.1107/S1600536813006739

**Published:** 2013-03-16

**Authors:** Malahat M. Kurbanova, Elnur Z. Huseynov, Atash V. Gurbanov, Abel M. Maharramov, Reza Kia

**Affiliations:** a Department of Organic Chemistry, Baku State University, Baku, Azerbaijan; b Department of Chemistry, Science and Research Branch, Islamic Azad University, Tehran, Iran; c Structural Dynamics of (Bio)Chemical Systems, Max Planck Institute for Biophysical Chemistry, Am Fassberg 11, 37077 Göttingen, Germany

## Abstract

In the title compound, C_21_H_24_BrNO_4_, the dihedral angle between the heterocyclic ring and the pendant aromatic ring is 80.20 (13)°. The hexahydroquinone [*i.e.* the one with the C=O group] ring adopts a sofa conformation. An intra­molecular O—H⋯O hydrogen bond generates an *S*(6) ring motif. The ethyl group is disordered over two sets of sites with a refined site occupancy ratio of 0.633 (10):0.366 (10). In the crystal, mol­ecules are linked by N—H⋯O inter­actions, forming chains parallel to [101]. There are no significant C—H⋯π or π–π inter­actions in the crystal structure.

## Related literature

For standard bond lengths, see: Allen *et al.* (1987 ▶). For hydrogen-bond motifs, see: Bernstein *et al.* (1995 ▶). For background to hexa­hydro­quinoline compounds and their applications, see: Sausins & Duburs (1988 ▶); Nakayama & Kasoaka (1996 ▶); Klusa (1995 ▶). For the synthesis of related compounds, see: Kumar *et al.* (2008 ▶); Song *et al.* (2012 ▶).
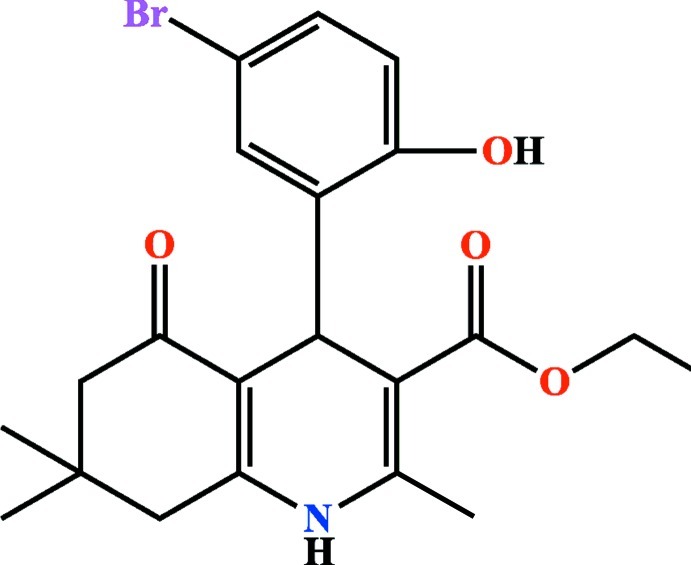


## Experimental

### 

#### Crystal data

C_21_H_24_BrNO_4_
*M* *_r_* = 434.32
Monoclinic, 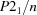

*a* = 9.5969 (3) Å
*b* = 19.0805 (5) Å
*c* = 11.0678 (3) Å
β = 97.387 (1)°
*V* = 2009.84 (10) Å^3^
*Z* = 4
Mo *K*α radiation
μ = 2.07 mm^−1^
*T* = 294 K
0.24 × 0.22 × 0.18 mm

#### Data collection

Bruker SMART APEXII CCD area-detector diffractometer
Absorption correction: multi-scan (*SADABS*; Bruker, 2005 ▶) *T*_min_ = 0.636, *T*_max_ = 0.707
23241 measured reflections
5008 independent reflections
3604 reflections with *I* > 2σ(*I*)
*R*_int_ = 0.022

#### Refinement

*R*[*F* ^2^ > 2σ(*F* ^2^)] = 0.050
*wR*(*F* ^2^) = 0.136
*S* = 1.05
5008 reflections
266 parameters
3 restraints
H-atom parameters constrained
Δρ_max_ = 1.10 e Å^−3^
Δρ_min_ = −1.02 e Å^−3^

### 

Data collection: *APEX2* (Bruker, 2005 ▶); cell refinement: *SAINT* (Bruker, 2005 ▶); data reduction: *SAINT*; program(s) used to solve structure: *SHELXTL* (Sheldrick, 2008 ▶); program(s) used to refine structure: *SHELXTL*; molecular graphics: *SHELXTL*; software used to prepare material for publication: *SHELXTL* and *PLATON* (Spek, 2009 ▶).

## Supplementary Material

Crystal structure: contains datablock(s) global, I. DOI: 10.1107/S1600536813006739/rz5051sup1.cif

Structure factors: contains datablock(s) I. DOI: 10.1107/S1600536813006739/rz5051Isup2.hkl

Supplementary material file. DOI: 10.1107/S1600536813006739/rz5051Isup3.cml

Additional supplementary materials:  crystallographic information; 3D view; checkCIF report

## Figures and Tables

**Table 1:** Hydrogen-bond geometry (Å, °)

*D*—H⋯*A*	*D*—H	H⋯*A*	*D*⋯*A*	*D*—H⋯*A*
O4—H4⋯O1	0.88	1.75	2.625 (3)	171
N1—H1⋯O2^i^	0.86	2.05	2.866 (3)	158

Symmetry code: (i) 
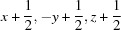
.

**Fig. 1.**
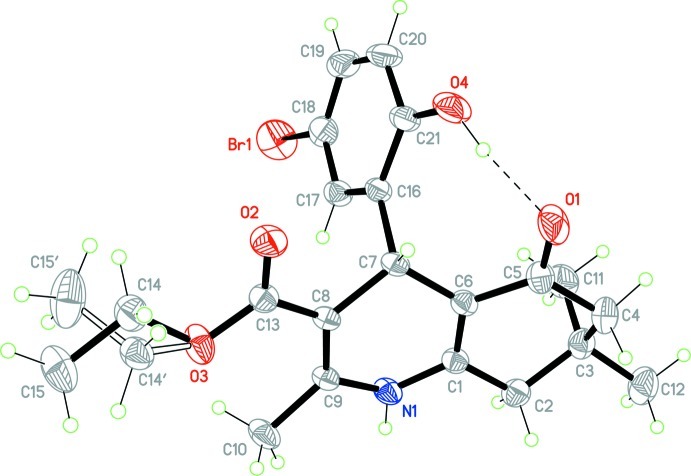
The molecular structure of the title compound, showing 40% probability displacement ellipsoids.

**Fig. 2.**
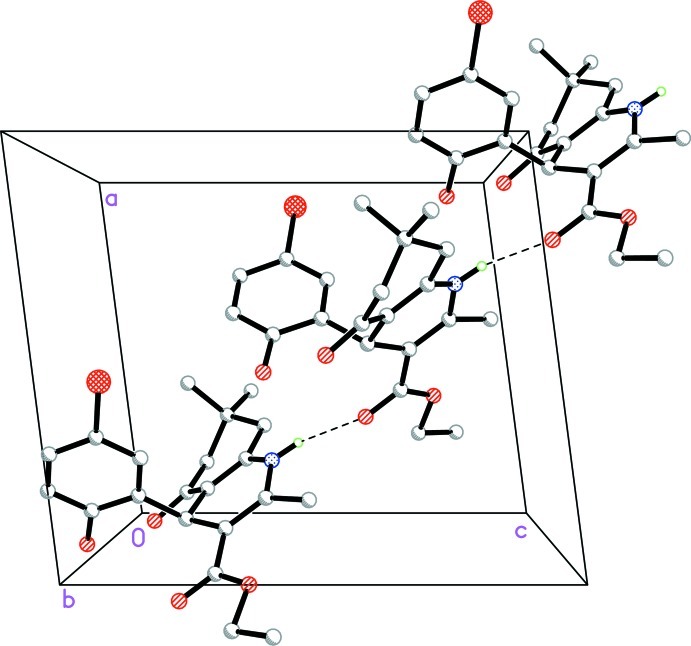
Packing diagram of the title compound viewed down the b axis, showing the linkage of molecules through N—H···O hydrogen interactions (dashed lines). Only H atoms involved in hydrogen bonding are shown.

## supplementary crystallographic information

### Comment

Hexahydroquinoline derivatives possess a variety of biological activities, such as vasodilatory, bronchodilatory, antiatherosclerotic, hepatoprotective, and antidiabetic activity (Sausins *et al.*, 1988), and some of them have been used as calcium channel modulators and curatives for cardiovascular diseases (Nakayama *et al.*, 1996). In past years, their uses as neuroprotectants, platelet anti-aggregatory agents, and cerebral anti-ischemic agents in the treatment of Alzheimer's disease and as chemosensitizers in tumor therapy have been also reported (Klusa, 1995).

The asymmetric unit of the title compound, Fig. 1, comprises a substituted hexahydroquinoline compound. Both six-membered rings of the hexahydroquinoline ring system adopt a half-boat conformation. Bond lengths (Allen *et al.*, 1987) and angles are within normal ranges. An intramolecular O—H···O hydrogen bond generates an *S(6)* ring motif (Bernstein *et al.*, 1995). In the crystal structure, molecules are linked together by intermolecular N—H···O hydrogen interactions (Table 1, Fig. 2) forming chains parallel to the [101] direction. The ethyl group is disordered over two sets of sites with a refined site occupancy ratio of 0.633 (10):0.366 (10). The compound contains one chiral center but the space group is centrosymmetric, so the molecule exists as a racemate.

### Experimental

5-Bromsalicylaldehyde (0.201 g, 1 mmol), ethyl acetoacetate (0.25 ml, 1 mmol), dimedone (0.14 g, 1 mmol), ammonium acetate (0.116 g, 1.5 mmol) and ethanol (15 ml) were charged in a round bottom flask. Then the reaction mixture was stirred at room temperature for 12 hours, then the product was separated by filtration. Recrystallization was effected by using ethanol as solvent. Yield 86%. M. p. 520 K.

### Refinement

The O- and N-bound H atoms were located in a difference Fourier map and constrained to ride on their parent atoms with O–H = 0.88 Å, N–H = 0.86 Å and U_iso_ (H) = 1.5 U_eq_(O, N). The C-bound H-atoms were included in calculated positions and treated as riding atoms with C–H = 0.93–0.98 Å and U_iso_(H) = 1.2 U_eq_(C) or 1.5 U_eq_(C) for methyl H atoms. Distance restraints were applied to the components of the disordered ethyl group.

### Crystal data

**Table:**

C_21_H_24_BrNO_4_	*F*(000) = 896
*M**_r_* = 434.32	*D*_x_ = 1.435 Mg m^−^^3^
Monoclinic, *P*2_1_/*n*	Melting point: 520 K
Hall symbol: -P 2yn	Mo *K*α radiation, λ = 0.71073 Å
*a* = 9.5969 (3) Å	Cell parameters from 2045 reflections
*b* = 19.0805 (5) Å	θ = 3.3–27.5°
*c* = 11.0678 (3) Å	µ = 2.07 mm^−^^1^
β = 97.387 (1)°	*T* = 294 K
*V* = 2009.84 (10) Å^3^	Block, colourless
*Z* = 4	0.24 × 0.22 × 0.18 mm

### Data collection

**Table:**

Bruker SMART APEXII CCD area-detector diffractometer	5008 independent reflections
Radiation source: fine-focus sealed tube	3604 reflections with *I* > 2σ(*I*)
Graphite monochromator	*R*_int_ = 0.022
φ and ω scans	θ_max_ = 28.3°, θ_min_ = 2.1°
Absorption correction: multi-scan (*SADABS*; Bruker, 2005)	*h* = −12→12
*T*_min_ = 0.636, *T*_max_ = 0.707	*k* = −25→25
23241 measured reflections	*l* = −14→14

### Refinement

**Table:**

Refinement on *F*^2^	Primary atom site location: structure-invariant direct methods
Least-squares matrix: full	Secondary atom site location: difference Fourier map
*R*[*F*^2^ > 2σ(*F*^2^)] = 0.050	Hydrogen site location: inferred from neighbouring sites
*wR*(*F*^2^) = 0.136	H-atom parameters constrained
*S* = 1.05	*w* = 1/[σ^2^(*F*_o_^2^) + (0.0542*P*)^2^ + 1.4854*P*] where *P* = (*F*_o_^2^ + 2*F*_c_^2^)/3
5008 reflections	(Δ/σ)_max_ = 0.001
266 parameters	Δρ_max_ = 1.10 e Å^−^^3^
3 restraints	Δρ_min_ = −1.02 e Å^−^^3^

### Special details

**Table:**

Geometry. All esds (except the esd in the dihedral angle between two l.s. planes) are estimated using the full covariance matrix. The cell esds are taken into account individually in the estimation of esds in distances, angles and torsion angles; correlations between esds in cell parameters are only used when they are defined by crystal symmetry. An approximate (isotropic) treatment of cell esds is used for estimating esds involving l.s. planes.
Refinement. Refinement of F^2^ against ALL reflections. The weighted R-factor wR and goodness of fit S are based on F^2^, conventional R-factors R are based on F, with F set to zero for negative F^2^. The threshold expression of F^2^ > 2sigma(F^2^) is used only for calculating R-factors(gt) etc. and is not relevant to the choice of reflections for refinement. R-factors based on F^2^ are statistically about twice as large as those based on F, and R- factors based on ALL data will be even larger.

### Fractional atomic coordinates and isotropic or equivalent isotropic displacement parameters (Å^2^)

**Table:**

	*x*	*y*	*z*	*U*_iso_*/*U*_eq_	Occ. (<1)
Br1	0.91668 (4)	0.11235 (2)	0.50601 (4)	0.09330 (19)	
O1	0.4895 (3)	0.41833 (11)	0.5435 (2)	0.0719 (6)	
O2	0.3062 (2)	0.18500 (11)	0.61555 (16)	0.0577 (5)	
O3	0.3634 (2)	0.13165 (11)	0.79229 (19)	0.0597 (5)	
O4	0.4398 (2)	0.31539 (12)	0.38649 (17)	0.0657 (6)	
H4	0.4496	0.3478	0.4434	0.099*	
N1	0.6847 (2)	0.28211 (11)	0.87199 (18)	0.0436 (5)	
H1	0.7348	0.2820	0.9422	0.052*	
C1	0.6826 (2)	0.34138 (12)	0.8040 (2)	0.0392 (5)	
C2	0.7744 (3)	0.40011 (14)	0.8550 (2)	0.0487 (6)	
H2A	0.8640	0.3812	0.8910	0.058*	
H2B	0.7312	0.4228	0.9193	0.058*	
C3	0.8001 (3)	0.45464 (14)	0.7599 (3)	0.0507 (6)	
C4	0.6576 (3)	0.47395 (14)	0.6893 (3)	0.0584 (7)	
H4A	0.6025	0.4986	0.7434	0.070*	
H4B	0.6726	0.5057	0.6238	0.070*	
C5	0.5754 (3)	0.41168 (14)	0.6362 (3)	0.0494 (6)	
C6	0.5958 (2)	0.34543 (12)	0.6974 (2)	0.0385 (5)	
C7	0.5182 (2)	0.28168 (12)	0.64397 (19)	0.0372 (5)	
H7A	0.4239	0.2969	0.6095	0.045*	
C8	0.5009 (2)	0.22835 (12)	0.74306 (19)	0.0350 (5)	
C9	0.5861 (2)	0.22942 (13)	0.85029 (19)	0.0379 (5)	
C10	0.5868 (3)	0.17973 (17)	0.9557 (2)	0.0576 (7)	
H10A	0.5907	0.1324	0.9270	0.086*	
H10B	0.5028	0.1862	0.9930	0.086*	
H10C	0.6674	0.1889	1.0144	0.086*	
C11	0.8970 (3)	0.42483 (18)	0.6729 (3)	0.0673 (8)	
H11A	0.8543	0.3841	0.6329	0.101*	
H11B	0.9855	0.4121	0.7182	0.101*	
H11C	0.9120	0.4596	0.6132	0.101*	
C12	0.8689 (4)	0.51939 (17)	0.8232 (4)	0.0734 (9)	
H12A	0.8085	0.5383	0.8779	0.110*	
H12B	0.8840	0.5540	0.7633	0.110*	
H12C	0.9574	0.5065	0.8684	0.110*	
C13	0.3830 (2)	0.18055 (13)	0.7107 (2)	0.0407 (5)	
C14	0.2502 (7)	0.0825 (4)	0.7444 (6)	0.0617 (18)	0.633 (10)
H14A	0.2704	0.0612	0.6690	0.074*	0.633 (10)
H14B	0.1606	0.1066	0.7295	0.074*	0.633 (10)
C15	0.2477 (6)	0.0278 (4)	0.8428 (7)	0.080 (2)	0.633 (10)
H15A	0.1797	−0.0076	0.8157	0.120*	0.633 (10)
H15B	0.2229	0.0495	0.9154	0.120*	0.633 (10)
H15C	0.3389	0.0067	0.8599	0.120*	0.633 (10)
C14'	0.2263 (8)	0.0963 (5)	0.7967 (10)	0.051 (3)	0.367 (10)
H14C	0.1501	0.1219	0.7502	0.061*	0.367 (10)
H14D	0.2062	0.0910	0.8799	0.061*	0.367 (10)
C15'	0.2497 (11)	0.0255 (6)	0.7387 (15)	0.094 (5)	0.367 (10)
H15D	0.1635	−0.0007	0.7296	0.141*	0.367 (10)
H15E	0.3205	−0.0001	0.7899	0.141*	0.367 (10)
H15F	0.2798	0.0326	0.6602	0.141*	0.367 (10)
C16	0.5888 (3)	0.25025 (13)	0.5398 (2)	0.0407 (5)	
C17	0.6996 (3)	0.20358 (14)	0.5642 (2)	0.0459 (6)	
H17A	0.7331	0.1922	0.6444	0.055*	
C18	0.7603 (3)	0.17389 (15)	0.4702 (3)	0.0561 (7)	
C19	0.7115 (4)	0.18797 (18)	0.3507 (3)	0.0662 (9)	
H19A	0.7507	0.1661	0.2881	0.079*	
C20	0.6038 (4)	0.23495 (18)	0.3256 (2)	0.0643 (9)	
H20A	0.5706	0.2451	0.2448	0.077*	
C21	0.5431 (3)	0.26783 (15)	0.4182 (2)	0.0499 (6)	

### Atomic displacement parameters (Å^2^)

**Table:**

	*U*^11^	*U*^22^	*U*^33^	*U*^12^	*U*^13^	*U*^23^
Br1	0.0748 (3)	0.1056 (3)	0.1086 (4)	0.0222 (2)	0.0469 (2)	0.0037 (2)
O1	0.0849 (15)	0.0519 (12)	0.0703 (14)	0.0074 (11)	−0.0226 (12)	0.0177 (10)
O2	0.0550 (11)	0.0705 (13)	0.0419 (10)	−0.0123 (9)	−0.0152 (8)	0.0044 (9)
O3	0.0498 (11)	0.0594 (11)	0.0645 (12)	−0.0181 (9)	−0.0140 (9)	0.0208 (10)
O4	0.0744 (14)	0.0777 (14)	0.0404 (10)	−0.0011 (11)	−0.0102 (9)	0.0150 (10)
N1	0.0421 (10)	0.0523 (12)	0.0332 (9)	−0.0084 (9)	−0.0066 (8)	0.0074 (9)
C1	0.0378 (11)	0.0429 (12)	0.0373 (11)	−0.0015 (9)	0.0062 (9)	0.0009 (10)
C2	0.0483 (14)	0.0512 (15)	0.0459 (14)	−0.0081 (11)	0.0028 (11)	−0.0035 (11)
C3	0.0509 (14)	0.0430 (14)	0.0595 (16)	−0.0037 (11)	0.0123 (12)	−0.0006 (12)
C4	0.0626 (17)	0.0386 (14)	0.0738 (19)	0.0055 (12)	0.0078 (14)	0.0038 (13)
C5	0.0514 (15)	0.0432 (14)	0.0526 (15)	0.0085 (11)	0.0036 (12)	0.0053 (11)
C6	0.0399 (12)	0.0405 (12)	0.0349 (11)	0.0032 (9)	0.0040 (9)	0.0039 (9)
C7	0.0373 (11)	0.0447 (13)	0.0280 (10)	0.0021 (9)	−0.0024 (8)	0.0054 (9)
C8	0.0337 (10)	0.0426 (12)	0.0284 (10)	0.0016 (9)	0.0032 (8)	0.0042 (9)
C9	0.0373 (11)	0.0463 (13)	0.0298 (10)	−0.0021 (10)	0.0031 (8)	0.0047 (9)
C10	0.0631 (17)	0.0708 (18)	0.0352 (12)	−0.0208 (14)	−0.0084 (11)	0.0165 (12)
C11	0.0628 (18)	0.070 (2)	0.074 (2)	−0.0017 (15)	0.0281 (16)	0.0055 (16)
C12	0.075 (2)	0.0544 (18)	0.092 (2)	−0.0175 (16)	0.0133 (18)	−0.0061 (17)
C13	0.0382 (11)	0.0438 (13)	0.0388 (12)	0.0019 (10)	0.0002 (9)	0.0021 (10)
C14	0.061 (3)	0.065 (4)	0.055 (4)	−0.022 (3)	−0.004 (3)	0.005 (3)
C15	0.065 (3)	0.084 (4)	0.093 (5)	−0.018 (3)	0.012 (3)	0.028 (4)
C14'	0.047 (4)	0.062 (6)	0.044 (6)	−0.012 (4)	0.003 (4)	0.006 (4)
C15'	0.053 (5)	0.064 (7)	0.169 (16)	−0.009 (5)	0.025 (7)	−0.002 (8)
C16	0.0462 (13)	0.0448 (13)	0.0311 (11)	−0.0106 (10)	0.0046 (9)	0.0024 (10)
C17	0.0461 (13)	0.0529 (14)	0.0398 (12)	−0.0080 (11)	0.0102 (10)	0.0020 (11)
C18	0.0559 (15)	0.0575 (16)	0.0594 (17)	−0.0096 (13)	0.0248 (13)	−0.0029 (13)
C19	0.081 (2)	0.073 (2)	0.0509 (16)	−0.0240 (18)	0.0309 (15)	−0.0138 (15)
C20	0.081 (2)	0.083 (2)	0.0298 (12)	−0.0262 (18)	0.0102 (13)	−0.0010 (13)
C21	0.0578 (15)	0.0581 (16)	0.0328 (12)	−0.0163 (13)	0.0022 (10)	0.0049 (11)

### Geometric parameters (Å, º)

**Table:**

Br1—C18	1.907 (3)	C10—H10A	0.9600
O1—C5	1.238 (3)	C10—H10B	0.9600
O2—C13	1.208 (3)	C10—H10C	0.9600
O3—C13	1.329 (3)	C11—H11A	0.9600
O3—C14	1.481 (5)	C11—H11B	0.9600
O3—C14'	1.485 (7)	C11—H11C	0.9600
O4—C21	1.357 (4)	C12—H12A	0.9600
O4—H4	0.8798	C12—H12B	0.9600
N1—C1	1.357 (3)	C12—H12C	0.9600
N1—C9	1.381 (3)	C14—C15	1.512 (4)
N1—H1	0.8600	C14—H14A	0.9700
C1—C6	1.356 (3)	C14—H14B	0.9700
C1—C2	1.491 (3)	C15—H15A	0.9600
C2—C3	1.523 (4)	C15—H15B	0.9600
C2—H2A	0.9700	C15—H15C	0.9600
C2—H2B	0.9700	C14'—C15'	1.526 (5)
C3—C12	1.528 (4)	C14'—H14C	0.9700
C3—C4	1.530 (4)	C14'—H14D	0.9700
C3—C11	1.531 (4)	C15'—H15D	0.9600
C4—C5	1.503 (4)	C15'—H15E	0.9600
C4—H4A	0.9700	C15'—H15F	0.9600
C4—H4B	0.9700	C16—C17	1.386 (4)
C5—C6	1.436 (3)	C16—C21	1.402 (3)
C6—C7	1.507 (3)	C17—C18	1.378 (4)
C7—C8	1.521 (3)	C17—H17A	0.9300
C7—C16	1.532 (3)	C18—C19	1.371 (4)
C7—H7A	0.9800	C19—C20	1.369 (5)
C8—C9	1.352 (3)	C19—H19A	0.9300
C8—C13	1.461 (3)	C20—C21	1.392 (4)
C9—C10	1.503 (3)	C20—H20A	0.9300
			
C13—O3—C14	111.3 (3)	C3—C11—H11A	109.5
C13—O3—C14'	123.0 (5)	C3—C11—H11B	109.5
C21—O4—H4	106.1	H11A—C11—H11B	109.5
C1—N1—C9	123.33 (19)	C3—C11—H11C	109.5
C1—N1—H1	118.0	H11A—C11—H11C	109.5
C9—N1—H1	116.5	H11B—C11—H11C	109.5
C6—C1—N1	119.6 (2)	C3—C12—H12A	109.5
C6—C1—C2	123.6 (2)	C3—C12—H12B	109.5
N1—C1—C2	116.8 (2)	H12A—C12—H12B	109.5
C1—C2—C3	113.1 (2)	C3—C12—H12C	109.5
C1—C2—H2A	109.0	H12A—C12—H12C	109.5
C3—C2—H2A	109.0	H12B—C12—H12C	109.5
C1—C2—H2B	109.0	O2—C13—O3	121.2 (2)
C3—C2—H2B	109.0	O2—C13—C8	122.4 (2)
H2A—C2—H2B	107.8	O3—C13—C8	116.37 (19)
C2—C3—C12	109.5 (2)	O3—C14—C15	105.0 (4)
C2—C3—C4	107.7 (2)	O3—C14—H14A	110.8
C12—C3—C4	110.2 (2)	C15—C14—H14A	110.8
C2—C3—C11	110.2 (2)	O3—C14—H14B	110.8
C12—C3—C11	109.1 (3)	C15—C14—H14B	110.8
C4—C3—C11	110.1 (3)	H14A—C14—H14B	108.8
C5—C4—C3	113.5 (2)	O3—C14'—C15'	102.1 (6)
C5—C4—H4A	108.9	O3—C14'—H14C	111.4
C3—C4—H4A	108.9	C15'—C14'—H14C	111.4
C5—C4—H4B	108.9	O3—C14'—H14D	111.4
C3—C4—H4B	108.9	C15'—C14'—H14D	111.4
H4A—C4—H4B	107.7	H14C—C14'—H14D	109.2
O1—C5—C6	121.2 (3)	C14'—C15'—H15D	109.5
O1—C5—C4	120.2 (2)	C14'—C15'—H15E	109.5
C6—C5—C4	118.6 (2)	H15D—C15'—H15E	109.5
C1—C6—C5	119.5 (2)	C14'—C15'—H15F	109.5
C1—C6—C7	120.9 (2)	H15D—C15'—H15F	109.5
C5—C6—C7	119.7 (2)	H15E—C15'—H15F	109.5
C6—C7—C8	110.56 (18)	C17—C16—C21	118.5 (2)
C6—C7—C16	111.54 (19)	C17—C16—C7	120.5 (2)
C8—C7—C16	112.36 (19)	C21—C16—C7	121.0 (2)
C6—C7—H7A	107.4	C18—C17—C16	120.4 (2)
C8—C7—H7A	107.4	C18—C17—H17A	119.8
C16—C7—H7A	107.4	C16—C17—H17A	119.8
C9—C8—C13	125.9 (2)	C19—C18—C17	121.5 (3)
C9—C8—C7	120.9 (2)	C19—C18—Br1	118.9 (2)
C13—C8—C7	113.17 (18)	C17—C18—Br1	119.6 (2)
C8—C9—N1	119.2 (2)	C20—C19—C18	118.7 (3)
C8—C9—C10	127.9 (2)	C20—C19—H19A	120.7
N1—C9—C10	112.93 (19)	C18—C19—H19A	120.7
C9—C10—H10A	109.5	C19—C20—C21	121.5 (3)
C9—C10—H10B	109.5	C19—C20—H20A	119.3
H10A—C10—H10B	109.5	C21—C20—H20A	119.3
C9—C10—H10C	109.5	O4—C21—C20	118.2 (2)
H10A—C10—H10C	109.5	O4—C21—C16	122.4 (2)
H10B—C10—H10C	109.5	C20—C21—C16	119.4 (3)
			
C9—N1—C1—C6	−11.6 (4)	C1—N1—C9—C8	14.5 (4)
C9—N1—C1—C2	166.2 (2)	C1—N1—C9—C10	−164.2 (2)
C6—C1—C2—C3	−19.6 (4)	C14—O3—C13—O2	6.0 (5)
N1—C1—C2—C3	162.7 (2)	C14'—O3—C13—O2	−21.9 (6)
C1—C2—C3—C12	168.6 (2)	C14—O3—C13—C8	−174.5 (4)
C1—C2—C3—C4	48.8 (3)	C14'—O3—C13—C8	157.6 (5)
C1—C2—C3—C11	−71.4 (3)	C9—C8—C13—O2	175.8 (2)
C2—C3—C4—C5	−53.9 (3)	C7—C8—C13—O2	−1.8 (3)
C12—C3—C4—C5	−173.3 (3)	C9—C8—C13—O3	−3.7 (4)
C11—C3—C4—C5	66.4 (3)	C7—C8—C13—O3	178.8 (2)
C3—C4—C5—O1	−152.6 (3)	C13—O3—C14—C15	176.1 (5)
C3—C4—C5—C6	29.2 (4)	C14'—O3—C14—C15	−62.6 (10)
N1—C1—C6—C5	169.3 (2)	C13—O3—C14'—C15'	102.7 (10)
C2—C1—C6—C5	−8.3 (4)	C14—O3—C14'—C15'	31.1 (9)
N1—C1—C6—C7	−9.4 (3)	C6—C7—C16—C17	84.8 (3)
C2—C1—C6—C7	173.0 (2)	C8—C7—C16—C17	−40.0 (3)
O1—C5—C6—C1	−174.8 (3)	C6—C7—C16—C21	−95.2 (3)
C4—C5—C6—C1	3.3 (4)	C8—C7—C16—C21	140.0 (2)
O1—C5—C6—C7	3.9 (4)	C21—C16—C17—C18	−1.8 (4)
C4—C5—C6—C7	−178.0 (2)	C7—C16—C17—C18	178.2 (2)
C1—C6—C7—C8	24.1 (3)	C16—C17—C18—C19	−1.6 (4)
C5—C6—C7—C8	−154.6 (2)	C16—C17—C18—Br1	177.85 (19)
C1—C6—C7—C16	−101.7 (2)	C17—C18—C19—C20	2.8 (4)
C5—C6—C7—C16	79.6 (3)	Br1—C18—C19—C20	−176.7 (2)
C6—C7—C8—C9	−21.1 (3)	C18—C19—C20—C21	−0.5 (5)
C16—C7—C8—C9	104.2 (2)	C19—C20—C21—O4	178.0 (3)
C6—C7—C8—C13	156.56 (19)	C19—C20—C21—C16	−2.8 (4)
C16—C7—C8—C13	−78.1 (2)	C17—C16—C21—O4	−177.0 (2)
C13—C8—C9—N1	−173.8 (2)	C7—C16—C21—O4	3.1 (4)
C7—C8—C9—N1	3.6 (3)	C17—C16—C21—C20	3.9 (4)
C13—C8—C9—C10	4.8 (4)	C7—C16—C21—C20	−176.0 (2)
C7—C8—C9—C10	−177.8 (3)		

### Hydrogen-bond geometry (Å, º)

**Table:**

*D*—H···*A*	*D*—H	H···*A*	*D*···*A*	*D*—H···*A*
O4—H4···O1	0.88	1.75	2.625 (3)	171
N1—H1···O2^i^	0.86	2.05	2.866 (3)	158

Symmetry code: (i) *x*+1/2, −*y*+1/2, *z*+1/2.
